# Knowledge and practice of childhood immunisation among parents in Kelantan, Malaysia: A cross-sectional study

**DOI:** 10.51866/oa.202

**Published:** 2024-01-31

**Authors:** Jamil Aiman Mohd Baharudin, Mohd Rizal Mohd Zain, Fahisham Taib, Intan Juliana Abd Hamid

**Affiliations:** 1 MD, MMed (Paediatric), Department of Paediatrics, School of Medical Sciences, Universiti Sains Malaysia, Kubang Kerian, Kelantan, Malaysia. Email: drrizal@usm.my; 2 MBBS, MMed (Paediatric), Faculty of Medicine and Health Sciences, Universiti Sains Islam Malaysia, Persiaran Ilmu, Bandar Baru Nilai, Nilai, Negeri Sembilan, Malaysia.; 3 MB BCh BAO, FRCPCH, Department of Paediatrics, Hospital Universiti Sains Malaysia, Kubang, Kerian, Kelantan, Malaysia.; 4 MD, MMed (Paediatric), PhD, Department of Clinical Medicine, Institut Perubatan dan Pergigian, Termaju, Universiti Sains Malaysia, Kepala Batas, Pulau Pinang, Malaysia.

**Keywords:** Immunization, Health knowledge, Attitude, Practice

## Abstract

**Introduction:**

Parents are key decision-makers in the immunisation practice and compliance of children. This study aimed to determine the knowledge and practice of immunisation among parents in Kelantan, Malaysia, and their associated factors.

**Methods:**

A cross-sectional study was conducted using a validated online questionnaire from May to June 2021. An invitation was distributed to parents attending a university hospital and extended families of staff through online platforms. A total of 311 parents participated in the study. The questionnaire consisted of 10 questions each on knowledge and practice and three questions on vaccination status. Descriptive analysis was performed. The associations between the sociodemographic characteristics and knowledge and practice scores were determined using the chi-square test, and predictive factors were identified using logistic regression analysis.

**Results:**

Most respondents were Malay (94.2%), Muslim (94.5%), women (79.7%) and married (96.1%). The median score for immunisation knowledge and practice was 8 (interquartile range [IQR]=2) and 7 (IQR=3), respectively. Multiple logistic regression revealed that parents who were unmarried or single, less educated, and had lower incomes were predicted to have poor knowledge of childhood vaccination (P<0.05). Conversely, those living outside Kota Bharu, less educated, and younger parents were predicted to have poor vaccination practice of childhood vaccination (P<0.05). Most respondents (97.8%) indicated completing their children’s vaccination schedule.

**Conclusion:**

Parental education and household income are associated with immunisation knowledge and practice. Improving access to information about childhood vaccination among targeted groups may further boost immunization coverage.

## Introduction

According to the World Health Organization, currently available vaccines protect against more than 20 vaccine-preventable diseases and prevent up to 3 million deaths each year. Apart from the apparent health benefits, other positive outcomes associated with immunisation include reduced antibiotic-resistant bacterial strains among the general population, extended life expectancy and enhanced economic growth by maintaining a healthy society.^1^

Since the inception of the Malaysian National Immunisation Programme in the 1950s, good compliance and immunisation coverage of more than 95% have resulted in a good health status among Malaysian children.^2^ However, reduced vaccination uptake in Malaysian children has been identified, and the rates have varied among states in the country.^3,4^ Kelantan has recorded the lowest childhood vaccination uptake at 72%, compared with the national rate at 95% in 2019.^5^ This reduction in vaccination uptake is worrying, as it may lead to a resurgence of diseases such as measles, diphtheria and pertussis. An uncontrolled spread of these diseases would devastatingly impact not only individuals’ health and economic status but also the community and country.

With the emergence of the COVID-19 pandemic in 2020, childhood immunisation practice has faced a new challenge. In a previous study, nearly a quarter of Saudi Arabian parents had a significant delay of more than 1 month in having their children vaccinated according to schedule, with 60% of respondents citing fear of being infected with COVID-19 as the reason for delay.^6^ This pattern was similarly observed in the United States, England and Pakistan in other studies.^7-9^

The causes of reduced vaccination uptake include vaccine hesitancy, refusal, default and misinformation.^10-13^ A study conducted in Selangor, Malaysia, demonstrated that the prevalence of vaccine defaulters was 20.7% and that individuals who had a lower educational level and a higher number of children tended to default. 10 The majority of earlier studies have discovered a significant connection between parental knowledge and vaccination habits and childhood immunisation coverage.^12,14^ It is hoped that by strengthening parental vaccination awareness, misconceptions will be eliminated, and vaccination uptake will improve.

Limited Malaysian studies have investigated parents’ knowledge, attitude and practice regarding childhood immunisation and their relationship with socio-demographic factors such as sex, educational level and socioeconomic status. Such studies have been mainly conducted in the Malaysian states of Pahang, Selangor and Kedah.^15-17^ There are no available data in Kelantan, despite it having a lower percentage of vaccinated children than other states.^5^ Accordingly, this study aimed to investigate the knowledge and practice of vaccination and their associated factors in a local Kelantanese population.

## Methods

We performed a cross-sectional study from May to June 2021, enrolling 311 respondents. Parents (either mother or father) who resided in Kelantan, consented to participate and had at least one child aged less than 2 years were included. Kelantanese parents who resided outside the state, were illiterate or unable to comprehend the Malay language and were non-biological parents were excluded. This study was approved by the Human Research Ethics Committee (USM/JEPeM/21030281).

The sample size was calculated using the OpenEpi sample size calculator version 3. The proportion of parents with good knowledge in Sungai Petani, Kedah, was 40%.^17^ Based on these figures and using the single-proportion formula, we calculated a sample size of 308, with a 20% dropout rate.

We utilised a validated questionnaire taken from the study by Awadh et al. in Bahasa Malaysia that was uploaded to Google Forms.^15^ A link to the questionnaire was generated in the form of a URL and QR code, which was shared via online platforms such as WhatsApp, Telegram, Facebook and Twitter. The convenience sampling method was used, whereby the link was distributed among the parents of inpatients or outpatients at the paediatric ward or paediatric clinic of the university hospital. The link was also forwarded to staff members of the paediatric department of the university hospital to increase the number of respondents. These staff members were asked to snowball forward the invitation to their families, friends and extended contacts. The online questionnaire was administered anonymously, and participation was voluntary.

The questionnaire consisted of three parts: Part one assessed parental socio-demographic characteristics including sex, age, marital status, number of children, race, religion, highest educational level, current employment status and total household income. Based on the total household income, participants were then classified to the top 20% (T20), middle 40% (M40) and bottom 40% (B40) in accordance with the Khazanah Research Institute guidelines.^18^

Part two consisted of a questionnaire on immunisation knowledge and practice that has been validated by Awadh et al.^15^ The questionnaire consists of 10 questions on knowledge and 10 questions on practice, with a Cronbach’s alpha value of 0.739 and 0.732, respectively, indicating good reliability of both subscales. For the knowledge questions, there are three answer options — yes, no and unsure. For the practice questions, yes/no options are used.

Part three comprised questions about the vaccination status of children and immunisation practice during the COVID-19 pandemic and the Movement Control Order (MCO) — a nationwide cordon sanitaire implemented by the federal Malaysian government beginning March 2020. When there was a delay in vaccinating their children, the respondents were asked to provide a reason.

Scoring for the knowledge and practice questions is determined by giving 1 point for each correct answer option and 0 points for either the incorrect, unsure or no response answer option. The maximum possible score is 10 for each category. Based on Bloom’s original cut-off points, both knowledge and practice scores were categorised as good (≥80%), moderate (60%–79%) or poor (≤59%).^19^

Descriptive analysis was conducted using SPSS version 27 (IBM, Chicago, IL). Categorical variables were presented as frequencies and percentages and numerical variables as medians with interquartile ranges (IQRs), as the data were non-normally distributed. The chi-square test was used to identify the association between the exposure (parents’ age, household income, number of children, area of residence, sex, marital status, race, religion, employment status and educational level) and outcome (knowledge and practice scores).

Simple and multiple binary logistic regression analyses were performed to compare subgroups within variables and predict their effect on the total knowledge and practice scores. Backward logistic regression was applied, and only variables that were included in the final model were presented.

Associations between the exposure and outcome were analysed using the chi-square test. All variables with significant results (P<0.05) in the bivariate analysis were included in the simple binary logistic regression analysis. For the simple binary logistic regression analysis, the significance level was pre-set at P<0.25. Any variables with P<0.25 were subsequently included in the multiple binary logistic regression analysis. For the multiple binary regression analysis, the significance level was pre-set at P<0.05. The findings of the multiple regression analysis were presented as beta coefficients, odds ratios (adjusted beta coefficients) with 95% confidence intervals and P-values.

## Results

The socio-demographic characteristics of the 311 participants are summarised in Table 1. Most participants were Malay (94.2%), Muslim (94.5%), women (79.7%) and married (96.1%). The majority were aged 26–35 years (60.1%), and slightly more than half of them had either one or two children (53.1%). Two-thirds of the participants had received education up to tertiary level, while 68.2% were employed.

**Table 1:** Socio-demographic characteristics (N=311).

Characteristics	Value	n (%)
Residence	Kota Bharu	129 (41.5)
	Outside Kota Bharu	182 (58.5)
Age	18–25 years	57 (18.3)
	26–35 years	187 (60.1)
	36–45 years	65 (20.9)
	46–60 years	2 (0.6)
Sex	Male	63 (20.3)
	Female	248 (79.7)
Marital status	Married	299 (96.1)
	Single	12 (3.9)
Number of children	1-2	165 (53.1)
	3-4	113 (36.3)
	>4	33 (10.6)
Race	Malay	293 (94.2)
	Non-Malay	18 (5.8)
Religion	Islam	294 (94.5)
	Non-Muslim	17 (5.5)
Educational level	Tertiary education	207 (66.6)
	Non-tertiary education	104 (33.4)
Employment status	Employed	212 (68.2)
	Unemployed	99 (31.8)
Household income	<RM 3030 (Bottom 40%)	127 (40.8)
	RM 3031-6619 (Middle 40%)	107 (34.4)
	>RM 6620 (Top 20%)	77 (24.8)

Overall, the knowledge on childhood immunisation among the parents in Kelantan was good, with a median score of 8 (IQR=2), while the practice of childhood immunisation was moderate, with a median score of 7 (IQR=3). Table 2 shows that 34.4% of the parents living in Kota Bharu had good knowledge of childhood immunisation as compared with 31.2% of those living in other districts. The 26–35-year age group had good immunisation knowledge compared with the other age groups (P<0.001). The male and female parents did not exhibit any significant difference in knowledge (P=0.26). Conversely, the married parents had significantly better knowledge than the single parents (P<0.001). There was no significant difference in the categorical knowledge scores between the Malays and non-Malays (P=0.40) and between the Muslims and non-Muslims (P=0.46). The number of participants with correct responses for the knowledge and practice questions is shown in Supplementary Tables 1 and 2, respectively.

**Table 2:** Knowledge level according to the socio-demographic characteristics (N=311).

		Knowledge level			
Variables		Poor n (%)	Moderate n (%)	Good n (%)	P-value
Residence	Kota Bharu	3 (1.0)	19 (6.1)	107 (34.4)	<0.001^*^
	Outside Kota Bharu	15 (4.8)	70 (22.5)	97 (31.2)	
Age	18–25 years	12 (3.9)	24 (7.7)	21 (6.8)	<0.001^*^
	26–35 years	4 (1.3)	51 (16.4)	132 (42.4)	
	36–45 years	2 (0.6)	12 (3.9)	51 (16.4)	
	46–60 years	0 (0.0)	2 (0.6)	0 (0.0)	
Sex	Male	1 (0.3)	20 (6.4)	42 (13.5)	0.26
	Female	17 (5.5)	69 (22.2)	162 (52.1)	
Marital status	Married	15 (4.8)	81 (26)	203 (65.3)	<0.001^*^
	Single	3 (1.0)	8 (2.6)	1 (0.3)	
Number of children	1-2	15 (4.8)	53 (17.0)	97 (31.2)	0.01^*^
	3-4	2 (0.6)	27 (8.7)	84 (27.0)	
	>4	1 (0.3)	9 (2.9)	23 (7.4)	
Race	Malay	18 (5.8)	85 (27.3)	190 (61.1)	0.40
	Non-Malay	0 (0.0)	4 (1.3)	14 (4.5)	
Religion	Islam	18 (5.8)	85 (27.3)	191 (61.4)	0.46
	Non-Muslim	0 (0.0)	4 (1.3)	13 (4.2)	
Educational level	Tertiary education	4 (1.3)	37 (11.9)	166 (53.4)	<0.001^*^
	Non-tertiary education	14 (4.5)	52 (16.7)	38 (12.2)	
Employment status	Employed	6 (1.9)	53 (17.0)	153 (49.2)	<0.001^*^
	Unemployed	12 (3.9)	36 (11.6)	51 (16.4)	
Household income	Bottom 40%	16 (5.1)	57 (18.3)	54 (17.4)	<0.001^*^
	Middle 40%	1 (0.3)	21 (6.8)	85 (27.3)	
	Top 20%	1 (0.3)	11 (3.5)	65 (20.9)	

* Significant (P<0.05)

More than half of the parents with tertiary education had good immunisation knowledge as compared with only 12.2% of those without tertiary education (P<0.001) (Table 3). Although the median score was the same (median=8, IQR=2), 49.2% of the employed parents and only 16.4% of the unemployed parents had good knowledge. For the household income, there was a significant difference in the total knowledge scores between the B40, M40 and T20 groups (P<0.001).

**Table 3:** Practice level according to the socio-demographic characteristics (N=311).

		Practice level			
Variables		Poor n (%)	Moderate n (%)	Good n (%)	P-value
Residence	Kota Bharu	11 (3.5)	31 (10.0)	87 (28.0)	<0.001^*^
	Outside Kota Bharu	66 (21.2)	63 (20.3)	53 (17.0)	
Age	18–25 years	28 (9.0)	20 (6.4)	9 (2.9)	<0.001^*^
	26–35 years	37 (11.9)	61 (19.6)	89 (28.6)	
	36–45 years	12 (3.9)	13 (4.2)	40 (12.9)	
	46–60 years	0 (0.0)	0 (0.0)	2 (0.6)	
Sex	Male	26 (8.4)	18 (5.8)	19 (6.1)	0.002^*^
	Female	51 (16.4)	76 (24.4)	121 (38.9)	
Marital status	Married	73 (23.5)	90 (28.9)	136 (43.7)	0.674
	Single	4 (1.3)	4 (1.3)	4 (1.3)	
Number of children	1-2	46 (14.8)	55 (17.7)	64 (20.6)	0.034^*^
	3-4	19 (6.1)	34 (10.9)	60 (19.3)	
	>4	12 (3.9)	5 (1.6)	16 (5.1)	
Race	Malay	75 (24.1)	89 (28.6)	129 (41.5)	0.276
	Non-Malay	2 (0.6)	5 (1.6)	11 (3.5)	
Religion	Islam	75 (24.1)	89 (28.6)	130 (41.8)	0.369
	Non-Muslim	2 (0.6)	5 (1.6)	10 (3.2)	
Educational level	Tertiary education	22 (7.1)	58 (18.6)	127 (40.8)	<0.001^*^
	Non-tertiary education	55 (17.7)	36 (11.6)	13 (4.2)	
Employment status	Employed	35 (11.3)	60 (19.3)	117 (37.6)	<0.001^*^
	Unemployed	43 (13.5)	34 (10.9)	23 (7.4)	
Household income	Bottom 40%	53 (17.0)	37 (11.9)	37 (11.9)	<0.001^*^
	Middle 40%	15 (4.8)	42 (13.5)	50 (16.1)	
	Top 20%	9 (2.9)	15 (4.8)	53 (17.0)	

* Significant (P<0.05)

Two-thirds of the parents living in Kota Bharu had good immunisation practice compared with only 29% of those living outside the capital city. The 26–35-year age group (28.6%) had good immunisation practice compared with the other age groups. While there was no difference in the knowledge scores between the sexes, the proportion of those with good practice scores was larger among the women (38.9%) than among the men (6.1%). There was no significant difference in the practice scores between the Malays and non-Malays and between the Muslims and non-Muslims.

The parents who received tertiary education had good immunisation practice compared with those who did not (P<0.001). The employed parents had good immunisation practice compared with the unemployed parents (P<0.001). There was also a significant difference in the practice scores between the household income groups (P<0.001).

**Figure 1:**
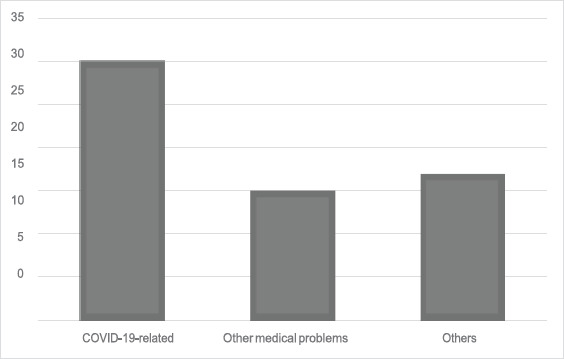
Reasons for not receiving vaccination on time (n=62).

Despite the implementation of the MCO, most parents (97.8%) reported that their children were fully vaccinated up to age, and only seven parents answered otherwise. However, about one-fifth of the children (n=62) did not receive their vaccinations on time owing to various reasons. These reasons are summarised in Figure 1. Thirty parents (48%) cited COVID-19-related reasons such as fear of contracting COVID-19, rescheduled appointments or quarantine on the date of vaccination. Fifteen children had a delay in receiving vaccination owing to non-COVID-related medical problems such as fever, upper respiratory tract infection or recent discharge from the ward. Other reasons were noted among the remaining 17 children, such as being unable to come to the clinic or forgetting their scheduled appointment.

Multiple logistic regression was performed to predict the total knowledge and practice scores according to the area of residence, age, marital status, number of children, educational level, employment status and household income (Tables 4 and 5). The parents who were unmarried or single, those who did not receive tertiary education and those with a lower household income were predicted to have poor knowledge on childhood vaccination (P<0.05). Conversely, the parents who were living outside Kota Bharu, those who did not receive tertiary education and those with younger age were predicted to have poor practice of childhood vaccination (P<0.05).

**Table 4:** Logistic regression analysis of the predictive factors of good knowledge scores (N=311).

Variables	Simple logistic regression		Multiple logistic regression	
	Crude OR (95% CI)	P-value	Adjusted OR (95% CI)	P-value
Living outside Kota Bharu	0.23 (0.13-0.40)	<0.001^*^	0.53 (0.28-1.02)	0.06
Having an older age	2.23 (1.50-3.32)	<0.001^*^	1.39 (0.86-2.23)	0.17
Being unmarried/divorced	0.04 (0.005-0.33)	0.003^*^	0.10 (0.01-0.86)	0.03^*^
Having more than two children	2.42 (0.51-11.42)	0.26		
Receiving non-tertiary education	0.14(0.08-0.24)	<0.001^*^	0.27 (0.13-0.57)	0.001^*^
Being unemployed	0.40 (0.24-0.67)	<0.001^*^	1.88 (0.89-3-97)	0.09
Having a higher household income	3.15(2.19-4.53)	<0.001^*^	1.77(1.09-4.59)	0.02^*^

* Significant (P<0.05). OR, odds ratio; CI, confidence interval

**Table 5:** Logistic regression analysis of the predictive factors of good practice scores (N=311).

Variables	Simple logistic regression		Multiple logistic regression	
	Crude OR (95% CI)	P-value	Adjusted OR (95% CI)	P-value
Living outside Kota Bharu	0.19(0.12-0.32)	<0.001^*^	0.42 (0.24-0.74)	0.003^*^
Having an older age	2.73 (1.84-4.06)	<0.001^*^	1.94 (1.20-3.14)	0.006^*^
Being unmarried/divorced	0.59(0.17-2.03)	0.41		
Having more than two children	2.19 (0.62-7.66)	0.21		
Receiving non-tertiary education	0.08 (0.04-0.17)	<0.001^*^	0.15 (0.07-0.35)	<0.001^*^
Being unemployed	0.24(0.14-0.42)	<0.001^*^	0.84 (0.40-1.75)	0.64
Having a higher household income	2.30(1.69-3.11)	<0.001^*^	0.92 (0.51-2.79)	0.70

* Significant (P<0.05). OR, odds ratio; CI, confidence interval

## Discussion

This study aimed to investigate vaccination knowledge and practice, along with their associated factors, in a local population in Kelantan. The findings showed that the median scores for immunisation knowledge and practice were 8 (IQR=2) and 7 (IQR=3), respectively. This finding suggests that parents in Kelantan have good knowledge but moderate adherence to childhood immunisation practice. The multiple logistic regression analysis revealed that the parents who were single, had a low educational level and had a low household income were more likely to have poor knowledge of childhood vaccination (P<0.05). Conversely, the parents who were residing in rural settings, had a low educational level and were younger were more likely to have poor practice of childhood vaccination (P<0.05).

Parents are the de facto decision-makers related to their children’s health and lives. Previous data have shown that the percentage of vaccinated children in Kelantan (72%) is lower than the national vaccination uptake in other states (>95%).^5^ Parental knowledge and practice of childhood immunisation have been reported to be influenced by sex, educational level and socio-economic status.^15-17^ In the present study, more than half of the respondents were aged 26-35 years and had either one or two children. About two-thirds received tertiary education and were employed. This sample bears resemblance to that of previous studies.^15,16^ The majority of the respondents in our study were women (79.7%), and this is probably reflective of how mothers are the primary caregivers in the local family unit and are directly responsible for the care of their children and their associated health issues, such as immunisation.^20^ The knowledge score is higher in our study (median=8, IQR=2) than in the studies by Awadh et al. (mean=6.84, standard deviation [SD] = 1.52) and Azmi et al. (mean=5.84, SD=2.26).^20,21^ In our study, the parents with tertiary education comprised a larger proportion of the sample. They were predicted to score better than those without a degree, consistent with previous reports showing that graduates score higher than non-graduates on knowledge tests regarding immunisation.^16,21-24^ Since the questionnaire was distributed via online platforms in this study, the respondents would need to possess a certain degree of literacy in information technology (IT) to participate. It can be postulated that being IT-proficient enables greater exposure and accessibility to information regarding vaccination, regardless of the educational level. This may explain the higher knowledge scores in the present study than in other studies in which respondents were interviewed face to face.

The total number of children in the family was found to be significantly related to the knowledge score in the present study. The parents with three to four children were more likely to have good knowledge than those with one or two children. This finding contradicts previous reports that the number of children is inversely associated with the knowledge score and that this is attributed to parents having less time to receive healthcare information when they have more children.^21,25^

In this study, the other significant predictors of knowledge among the parents were marital status, educational level and household income. The parents with a higher household income were more likely to have good knowledge, as those who earn more are also more likely to be better educated and have more access to resources on health information.^26^ Single parenting was a negative outcome predictor in our study. This could be attributed to the fact that the single parents in our study were young, did not receive tertiary education or had a lower household income - variables that were established to be associated with poorer immunisation knowledge. Sex, race and religion did not have any significant impact on the knowledge score, in line with previous reports.^15,20^

The demographic characteristics that were found to significantly predict immunisation practice were the area of residence, age and educational level. The parents living in Kota Bharu had better practice scores than those living elsewhere. This finding is in accordance with other reports in Malaysia, India and even developed countries such as Canada.^17,27,28^ Health practices have been shown to be better among urban populations owing to health services in general and vaccines specifically being more readily accessible in these areas.

The younger parents were predicted to have a significantly lower score than the parents older than 25 years in this study. In their respective studies, Al-Lela et al. and Hu et al. suggested that older parents tend to have better immunisation practice owing to having more experience and a better understanding of the national vaccine policy.^29^ Similarly, the parents who had more than two children were observed to have better practice scores in our study, again mirroring the findings of Hu et al.^29^

The most significant predictor of immunisation practice was the educational level. As with the knowledge score, the parents without tertiary education were more likely to score lower than those with college or university education. According to a previous study, graduate parents were almost four times more likely to vaccinate their children than non-graduate parents.^16^ A strong association between parents’ educational level and practice of vaccination was also found in studies performed in China and Pakistan.^29,30^

Despite movement restrictions and a reduced number of appointments per day in health clinics during the pandemic, most respondents (97.8%) showed perseverance in vaccinating their children as per the National Immunisation Programme. Sixty-two parents (19.9%) reported a delay in vaccinating their child, and only half of them cited COVID-related reasons as the cause. This is not far off from figures obtained from studies abroad indicating that the outlook and general attitude towards the pandemic are similar between parents in Malaysia and overseas.^6-9^

Several limitations exist in this study, with the first being the possibility of bias in sampling. With the national MCO in place, we decided to disseminate the questionnaire through online platforms and recruit participants via snowball convenience sampling. This introduced an element of bias, as new participants most likely came from a similar demographic background as the person recruiting them. Second, the subdivision of each demographic category was mostly dichotomous, leading to less distinction among the sample. Third, as this study involved sampling only in a single institution, the findings may not be generalised to the entire state of Kelantan or Malaysia.

## Conclusion and recommendation

There are still gaps in the socio-demographic characteristics that present a hindrance to achieving full immunisation coverage for children in Kelantan. Improving the educational level of parents while also addressing the income inequality between groups may result in better immunisation knowledge and practice. Establishing mobile immunisation services that specifically target rural households or families far from urban outreach may also help to increase vaccination uptake in Kelantan. Further, improving road infrastructures so that families living in rural areas have better connectivity to their nearest local clinic may facilitate good immunisation practice. These measures targeting susceptible groups of parents would make the greatest difference in improving the vaccination uptake among children in the developing country.

## Data sharing statement

Further information on the data can be requested via email to the corresponding author.

**How does this paper make a difference in general practice?**

- This study provides valuable insights into the current knowledge and practices among the population in Kelantan and can be used to compare with other regions in Malaysia.
- The findings of this study highlight the imperative for increased efforts to enhance awareness and understanding of immunization, particularly among parents with lower education levels.
- Additionally, it was observed that parents facing economic challenges, possessing lower educational backgrounds, and residing in rural areas exhibited suboptimal practices regarding immunization. Consequently, there is a need for primary care practitioners to prioritize and intensify efforts in these specific demographics.
